# Primary Adenosquamous Carcinoma of the Prostate

**DOI:** 10.3390/diagnostics14060645

**Published:** 2024-03-19

**Authors:** Roksolana Demianets, Dong Ren, Roozbeh Houshyar, Giovanna A. Giannico, Cary Johnson

**Affiliations:** 1 Department of Pathology and Laboratory Medicine, University of California, Irvine, CA 92868, USA; dren3@hs.uci.edu (D.R.);; 2 Department of Radiological Sciences, University of California, Irvine, CA 92868, USA

**Keywords:** prostate gland, adenocarcinoma, adenosquamous carcinoma

## Abstract

Prostate cancer accounts for 29% of malignant diagnoses among men in the United States and is the second leading cause of death from cancer. Effective screening methods and improved treatment have decreased the mortality rate significantly. This decreased mortality rate, however, does not apply to all histologic variants. Adenosquamous carcinoma of the prostate is an extremely aggressive neoplasm with no current known curative therapy. It is often diagnosed after chemotherapy, radiation, or androgen deprivation therapy for traditional prostatic adenocarcinomas. Primary carcinomas of the prostate with squamous features include, but are not limited to, pure squamous cell carcinoma and adenocarcinoma mixed with squamous cell carcinoma (SCC). Important distinguishable clinical features of adenosquamous carcinoma include normal prostate-specific antigen (PSA) levels, even with advanced disease and osteolytic versus osteoblastic metastatic lesions in adenocarcinoma. Additional entities to consider in the differential diagnosis are squamous metaplasia of the prostate, secondary involvement of pure SCC, and urothelial carcinoma with squamous differentiation. Here, we present a de novo case of adenosquamous carcinoma in a 48-year-old man who rapidly developed extensive metastatic disease.

A 48-year-old otherwise healthy male presented for his annual exam with complaints of newly developed nocturia, hesitancy, and a decreased urinary stream. The digital rectal exam was unremarkable. The PSA level at the time of presentation was elevated at 9.34 ng/mL (normal < 4 ng/mL). The patient was referred for magnetic resonance imaging (MRI) that demonstrated a large lesion with marked diffusion restriction, early enhancement centered in the right basal peripheral zone, extracapsular spread, involvement of the right neurovascular bundle at the base, and bilateral seminal vesicle invasion. The Prostate Imaging Reporting and Data System (PIRADS) was 5/5 (Figure 1). There was another wedge-shaped T2 hypointensive PIRADS 3/5 lesion in the left apical peripheral zone with early enhancement but equivocal appearance on diffusion-weighted images.

On subsequent testing, the PSA had increased to 13.9 ng/mL. The patient underwent MRI fusion biopsy with a diagnosis of prostatic adenocarcinoma, Gleason score 7 (4 + 3), Grade Group 3 in 5 out of 11 cores, and perineural invasion. A positron emission tomography (PET) scan and a computed tomography (CT) scan (PET/CT) showed abnormal increased radiotracer uptake within the posterior paramedian aspect of the prostate gland extending from the level of the base to the apex and demonstrating a maximum standardized uptake value (SUV) of 30, as well as seminal vesicle involvement, greater on the right. There was no evidence of distant metastatic disease (Figure 2). The decision was made to proceed with robotic radical prostatectomy and bilateral pelvic lymph node dissection. The final pathological diagnosis was prostatic adenocarcinoma with squamous differentiation, extraprostatic extension, seminal vesicle invasion, and lymphovascular and perineural invasion. The carcinoma had a 25% squamous component (Figure 3), a 25% pattern 4 glandular component, and a 50% pattern 5 component (Figure 4).

The squamous component was reactive to p40, p63, and cytokeratin 5/6. The cells with glandular epithelial differentiation expressed NKX 3.1, PSA, PSAP, and PIN4 (Figure 5).

Both cells’ populations were negative for uroplakin Il and III, PAX-8, GATA3, synaptophysin, chromogranin, and INSM1. Four months after the surgery, the patient presented with severe pain in the lumbar/coccygeal area with increased PSA levels up to 0.23 ng/mL. An MRI of the pelvis demonstrated a mass in the prostatectomy bed measuring 5.9 cm × 4.8 cm × 4.9 cm and pelvic lymphadenopathy that was concerning for metastatic disease (Figure 6).

A CT scan of the chest, abdomen, and pelvis revealed a newly developed 7.8 cm lesion of the right posterior eight rib with a soft tissue component, a right middle lobe lung nodule measuring 0.9 cm, and numerous hypo-enhancing bilobar liver lesions ranging from 0.5 to 1.4 cm (Figure 7).

The rib lesion was biopsied, and the histology was consistent with poorly differentiated carcinoma, with a morphology similar to the prostatectomy specimen (Figure 8). The cells stained positive for CK5/6 and p63, but were negative for NKX 3.1, PSA, and PSAP.

The patient was started on androgen deprivation therapy (ADT) and died 3 months after starting the treatment.

Adenosquamous prostate adenocarcinoma accounts for less than 1% of all prostate carcinomas [1]. Its etiology remains controversial. The two main theories suggest origin from squamous metaplasia as a result of chronic inflammation or pluripotent stem cells capable of multidirectional differentiation. Other theories include the collision of two separate tumors and clonal evolution of persistent carcinoma secondary to radiation or ADT, which are risk factors for secondary etiology [2], albeit not present in our case. Clinically, patients present with nonspecific symptoms such as nocturia, dysuria, hematuria, pelvic pain, urinary tract infections, or systemic symptoms, such as bone pain in cases of advanced disease. Laboratory testing often reveals normal levels of PSA and PSAP. Therefore, PSA might be an inadequate diagnostic biomarker for this malignancy [1]. Our patient had a low postsurgical PSA level considering the bulk of the recurrent disease on imaging. Some authors suggest that a loss of PSA secretion may lead to a lack of response to androgen blockage therapy [3].

Imaging overall cannot contribute to differential diagnosis except for in advanced cases, when bone scans typically reveal osteolytic lesions as opposed to osteoblastic, which is typical of conventional adenocarcinoma. Our patient had osteolytic rib metastasis. Metastatic lesions have been described in the peritoneum, diaphragm, liver, and lungs. Adenosquamous components are generally not PSMA-avid, and FDG PET can be considered to assess the disease burden [4]. Histologic examination provides a reliable means for the diagnosis of adenosquamous prostatic carcinoma. Histologically, the presence of both glandular and squamous components is pathognomonic. The percentage of squamous components ranges from 5% to 95%. Typical squamous components will demonstrate keratinization, squamous pearls, and intercellular bridges. Squamous origin can be confirmed with p63, p40, and CK5/6 positivity on immunohistochemistry. A poorly differentiated squamous cell component, which might show the above-mentioned features, may be more difficult to diagnose. In case both glandular and squamous components are present, a Gleason score must be given only for the glandular component [5].

The metastatic involvement of the prostate must be excluded. The morphologic and immunohistochemical pictures along with the clinical findings, presenting symptomatology and radiological findings, are important in this distinction. Specifically, immunohistochemical stains such as GATA3 and uroplakin II and III may be helpful to exclude urothelial origin. Due to its rarity, the knowledge of the molecular profile in adenosquamous morphology is limited. Rearrangement involving ERG and BRAF are the most common among both secondary and de novo cases. The frequency of these rearrangements is similar, however, to that seen in conventional adenocarcinoma. It is recommended to perform NGS (next-generation sequencing) and FISH (fluorescence in situ hybridization) in addition to immunohistochemistry, as the latter is less sensitive for adenosquamous components [6].

Currently, there is no definitive treatment. Therapy depends on the individual case and ranges from radiation to surgery (radical prostatectomy) to hormonal therapy and combinations of the aforementioned. Generally, surgical intervention is limited to patients with localized disease [2]. ADT is for patients with non-localized disease. Some authors have hypothesized that the response to ADT is poor when there is a predominant squamous carcinoma component, so some oncologists will provide hormonal therapy only for adenocarcinoma-predominant cases [1]. Overall, hormonal and radiation therapy are used for more advanced stages. Unfortunately, our case demonstrated no long-term benefit from combined surgical, ADT, chemotherapy, and radiation therapy, most likely due to the extensive metastatic disease.

The prognosis for adenosquamous prostatic carcinoma is poor, with a median survival of 12–14 months. Many cases in the literature were already metastatic at diagnosis. Metastatic cases had only 20% survival rates at six months, with all patients dying within one year of diagnosis. Nonetheless, the overall survival was higher among patients undergoing surgery compared to patients who did not have surgical treatment; however, this may be due to less-advanced disease [7].

The parameters of adenosquamous carcinoma of the prostate in the existing literature, including age, PSA level, Gleason score, grading, metastatic status, treatment and survival, are summarized in Table 1. Our patient was 48 years old at the time of presentation, while the median age of presentation in previously described cases of adenosquamous prostate cancer is 65. The Gleason score in those patients varies from 6 to 10. Treatment includes a combination of radiation, ADT, chemotherapy, and diethylstilbestrol. The majority of patients develop metastasis (most commonly in the lungs and bones). The overall survival of our patient was 8 months, while survival in the reported literature varies from 1 month to 13 months. Multiple factors, including young age, extensive squamous components, high Gleason scores, and seminal vesicle invasion, could contribute to a low survival rate in our case.

In conclusion, adenosquamous carcinoma of the prostate is an aggressive malignancy with poor prognosis and no effective curative treatment to date. This case re-emphasizes the need for further investigation of the etiology, molecular characteristics, and diagnostic approach in order to establish the optimal management of this entity.

## Figures and Tables

**Figure 1:**
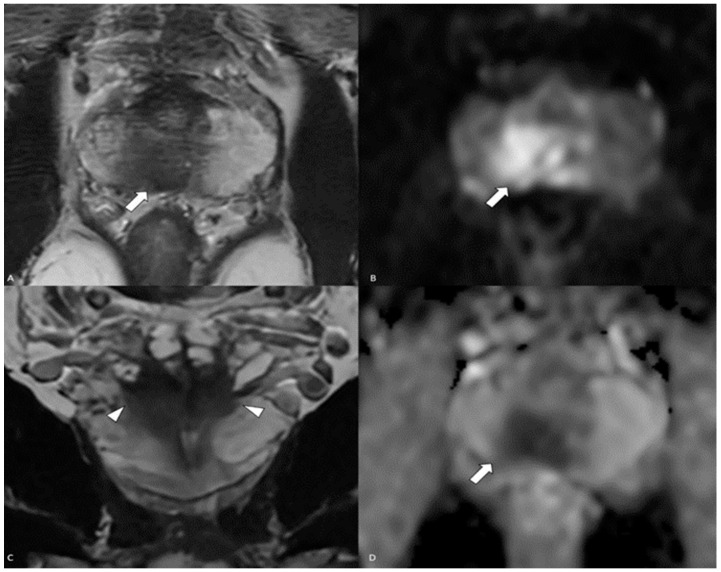
MRI of the prostate: (**A**) axial T2 demonstrates a posterior peripheral-zone T2-hypointense lesion (white arrow) with (**B**,**D**) restricted diffusion (white arrows), and (**C**) coronal T2 demonstrates seminal vesicle invasion (white arrowheads).

**Figure 2:**
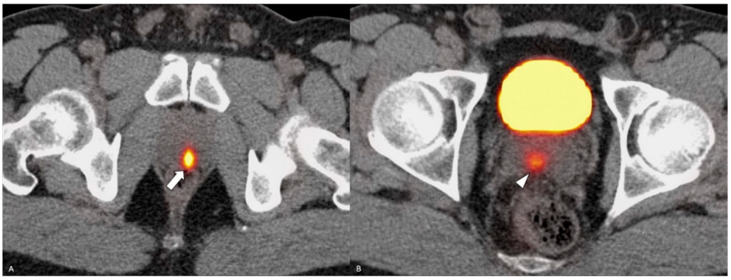
PSMA PET/CT. Part (**A**)—radiotracer uptake is demonstrated in the posterior prostate (white arrow). Part (**B**)—tumor is extending into the seminal vesicles (white arrowhead).

**Figure 3:**
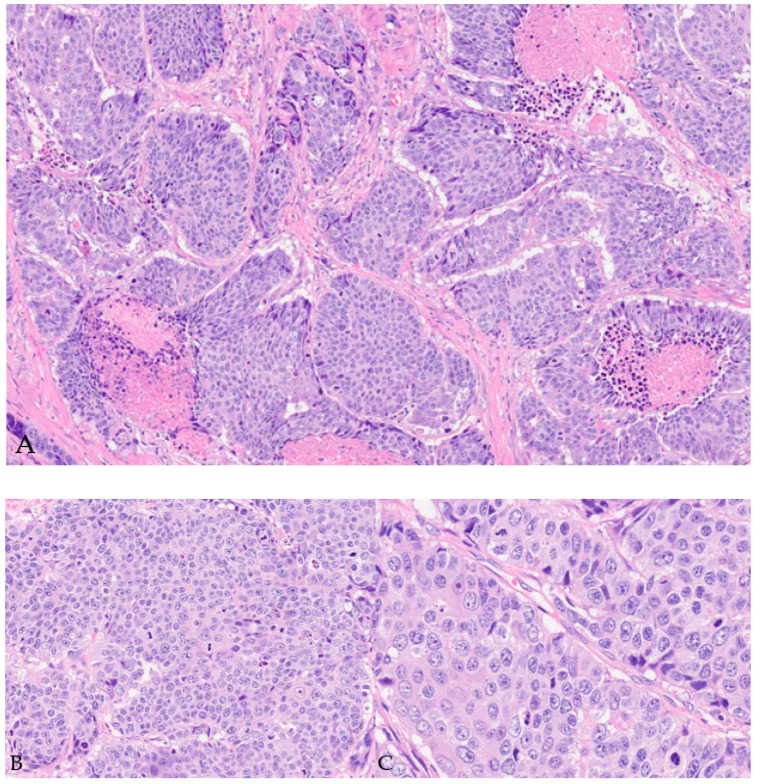
Microscopic examination of prostatectomy specimen (**A**,**B**). H&E staining image with extensive squamous component. (Magnification: 10× (**A**), 20× (**B**) and 40× (**C**)).

**Figure 4:**
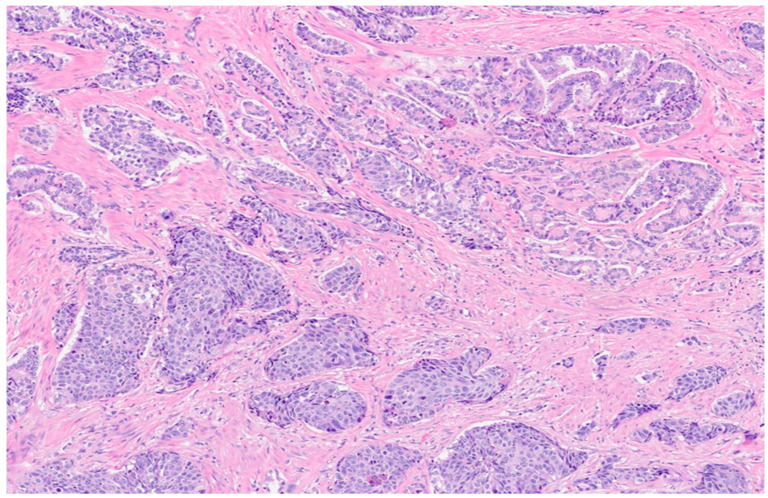
Microscopic examination of prostatectomy specimen. H&E staining image showing the concomitant presence of traditional adenocarcinoma (magnification: 4×).

**Figure 5:**
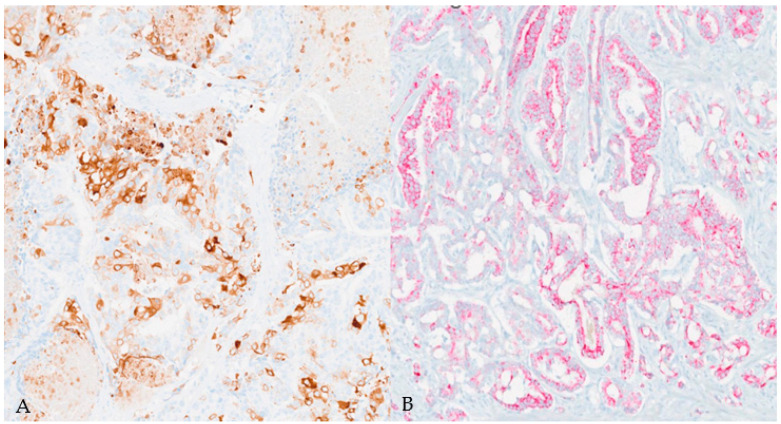
Immunohistochemical findings in squamous ((**A**), CK5/6 stain) and adenocarcinoma ((**B**), PIN4 stain) components. (Magnification: 10×.)

**Figure 6:**
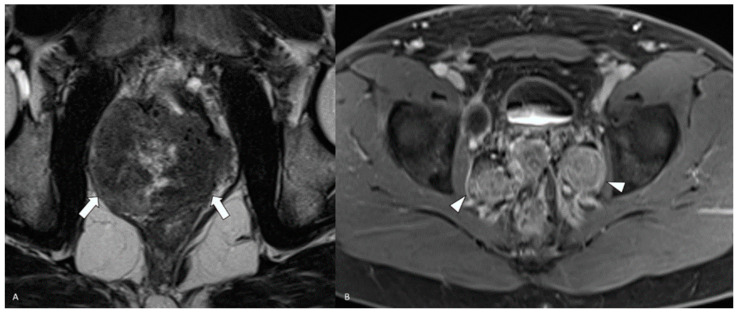
MRI of the pelvis: (**A**) axial T2 demonstrates a large heterogeneously hypointense lesion in the prostatic bed (white arrows). (**B**) Axial contrast-enhanced T1 fat-saturated sequence shows bilateral pelvic adenopathy (white arrowheads).

**Figure 7:**
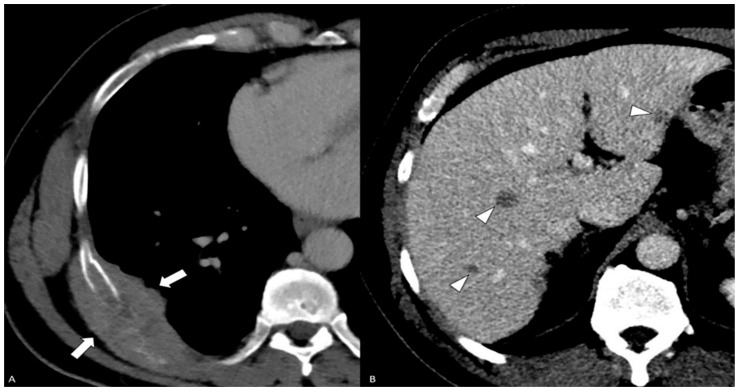
Contrast-enhanced CT—(**A**) axial post-contrast CT of the chest shows an expansile right 8th rib mass with a subpleural component (white arrows). (**B**) Axial post-contrast CT of the abdomen shows multiple small ill-defined liver lesions (white arrowheads).

**Figure 8:**
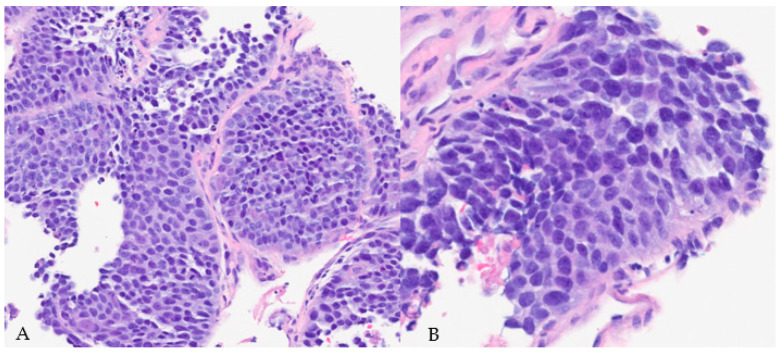
H&E staining image of core-needle biopsy of the rib lesion demonstrating sheets of hyperchromatic and pleomorphic cells with prominent nucleoli, frequent mitosis, and poorly differentiated morphology (magnification: 20× (**A**) and 40× (**B**)).

**Table 1:** Summary of publications on adenosquamous carcinoma of the prostate. ADT: androgen deprivation therapy; AWND: alive with no disease; Chemo: chemotherapy; DD: died of disease; DES: diethylstilbestrol; Met: metastasis; N/A: not/available; Ra: radiation.

Study	Age	PSA Level (ng/mL)	Gleason Score	Grade Group	Met	Treatment	Follow-Up (Months)	Outcome
Accetta/1983 [8]	77	N/A	N/A	III/IV	manubrium, bone (T-11)	Ra, (DES)	N/A	N/A
Devaney/1991 [9]	70	N/A	N/A	III	No	DES	N/A	N/A
Kitamura/2021 [10]	76	13.37	5 + 4 = 9 and 5 + 5 = 10	V	lung, bone (ischium)	ADT, chemo	13	DD
Azzi/2022 [1]	62	3.11	3 + 3 = 6 (<10%)	N/A	rectum, lung	Ra, chemo	9	AWND
Mishra/2014 [2]	60	2.14	N/A	N/A	bone	ADT	9	DD
Hennessey/2019 [11]	66	12.7	5 + 5 = 10	IV/V	bladder	ADT, Ra, chemo	20	AWND
Egilmez/2005 [12]	58	12	2 + 1 = 3 (<10%)	N/A	No	ADT	1	DD
Bassler/1999 [13]	55	8.5	4 + 3 = 7	N/A	No	ADT, Ra	N/A	N/A
Singh/2013 [5]	65	1.07	N/A	N/A	No	Ra, chemo	1	DD
Yang/2024 [14]	57	4.96	5 + 4 = 9	V	Lung, bone, lymph nodes	ADT, Ra	N/A	N/A

## Data Availability

Not applicable.
